# Temporal Changes of Protein Composition in Breast Milk of Chinese Urban Mothers and Impact of Caesarean Section Delivery

**DOI:** 10.3390/nu8080504

**Published:** 2016-08-17

**Authors:** Michael Affolter, Clara L. Garcia-Rodenas, Gerard Vinyes-Pares, Rosemarie Jenni, Iris Roggero, Ornella Avanti-Nigro, Carlos Antonio de Castro, Ai Zhao, Yumei Zhang, Peiyu Wang, Sagar K. Thakkar, Laurent Favre

**Affiliations:** 1 Nestlé Research Center, Nestec Ltd., Lausanne 1000, Switzerland; clara.garcia@rdls.nestle.com (C.L.G.-R.); rosemarie.jenni@rdls.nestle.com (R.J.); iris.roggero@rdls.nestle.com (I.R.); ornella.avanti-nigro@rdls.nestle.com (O.A.-N.); carlosantonio.decastro@rdls.nestle.com (C.A.d.C.); sagar.thakkar@rdls.nestle.com (S.K.T.); laurent.favre1@rdls.nestle.com (L.F.); 2 Nestlé Research Center Beijing, Nestec Ltd., Beijing 100095, China; gerard.vinyespares@rd.nestle.com; 3 Department of Nutrition and Food Hygiene, School of Public Health, Peking University, Beijing 100191, China; xiaochaai@163.com (A.Z.); zhangyumei@hsc.pku.edu.cn (Y.Z.); 4 Department of Social Medicine and Health Education, School of Public Health, Peking University, Beijing 100191, China; wpeiyu@bjmu.edu.cn

**Keywords:** breast milk, proteins, immune factors, Chinese mothers, CAESAREAN-section

## Abstract

Human breast milk (BM) protein composition may be impacted by lactation stage or factors related to geographical location. The present study aimed at assessing the temporal changes of BM major proteins over lactation stages and the impact of mode of delivery on immune factors, in a large cohort of urban mothers in China. 450 BM samples, collected in three Chinese cities, covering 8 months of lactation were analyzed for α-lactalbumin, lactoferrin, serum albumin, total caseins, immunoglobulins (IgA, IgM and IgG) and transforming growth factor (TGF) β1 and β2 content by microfluidic chip- or ELISA-based quantitative methods. Concentrations and changes over lactation were aligned with previous reports. α-lactalbumin, lactoferrin, IgA, IgM and TGF-β1 contents followed similar variations characterized by highest concentrations in early lactation that rapidly decreased before remaining stable up to end of lactation. TGF-β2 content displayed same early dynamics before increasing again. Total caseins followed a different pattern, showing initial increase before decreasing back to starting values. Serum albumin and IgG levels appeared stable throughout lactation. In conclusion, BM content in major proteins of urban mothers in China was comparable with previous studies carried out in other parts of the world and C-section delivery had only very limited impact on BM immune factors.

## 1. Introduction

Evolution has shaped human breast milk (BM) composition to protect the infant against disease(s) and to supply their nutritional needs [1]. BM proteins are one of the major contributors to this dual role in early infancy. BM proteins are the primary source of amino acids required for body protein building and can facilitate nutrient digestion as well as increase their bioavailability. BM proteins can also act as immunologically active molecules able to confer passive protection against pathogens, to stimulate the infant’s antimicrobial defences or to modulate the infant immune maturation and responses [2,3,4].

More than 2500 distinct protein sequences have been identified in BM [5]. The most abundant BM proteins include lactoferrin, α-lactalbumin, serum albumin and the β- and κ-casein fractions, collectively representing about 85% of total BM proteins [6]. Multiple biological activities have been proposed for lactoferrin, and possibly the best documented effect in the infants is protection against gastrointestinal infections [7]. Similarly, a multimeric α-lactalbumin-lipid complex (HAMLET) found in BM has potent pro-apoptotic effects on bacterial [8] and tumoral cells, while sparing healthy eukaryotic cells [9]. By contrast, serum albumin and caseins likely have a predominantly nutritional role as opposed to lactoferrin, and these proteins appear to be readily digested by the infant gastrointestinal proteases. Nevertheless, some biological activities have been proposed for the peptides produced during the digestion of these proteins [10]. For example, antibacterial activity has been found upon gastric digestion of β-casein in infants [11].

Immune factors are also important BM components, representing up to 10% of total proteins. Immunoglobulins (Ig) and members of the transforming growth factor (TGF)-β family are the most studied key partners of the immunological activity found in colostrum, transitional and mature milk, ensuring transfer of passive immunity from mother to offspring [12], as well as supporting the onset of gut homeostasis in the neonate [13,14,15,16,17]. IgA, or more precisely secretory IgA, is the major isotype found in BM, followed then by IgM and IgG. Its dynamic of secretion over lactation period has been investigated in several studies, showing high content in colostrum, followed by a rapid diminution during transition milk to then remain stable in mature milk [18,19]. The TGF-β family constitutes the most abundant cytokines of BM and consists of three isoforms, of which TGF-β2 predominates, followed by TGF-β1 [20]. Data on the changes of the secretion over lactation period of these two cytokines are more limited than for Igs, but tend to show overall similar patterns [21].

Infants born by Caesarean section (C-section) suffer from an associated increased risk of development later in life of immune-related diseases [22,23,24]. These alterations are commonly attributed to altered microbiota colonization patterns in those infants due to the absence of the initial inoculation of maternal vaginal and faecal microbiota [22]. However, potential impact of delivery mode on BM-related immune parameters may also be an important contributing factor. Indeed, data available from several studies indicate a delayed onset of lactation following C-section [25,26] preventing the new-born to gain prompt access to beneficial components of BM. In contrast, little is known about the impact of C-section delivery on BM composition and in particular on the milk immune factors. Current data from studies focusing on immunoglobulin content in colostrum samples do not allow us to draw a clear conclusion on a potential impact of the mode of delivery on the presence of these antibodies in the BM [4,27]. To our knowledge, no data are currently available on the effect of C-section delivery on major BM immune factors throughout transitional and mature milk.

Hence, the main objective of the present work was to assess the specific temporal changes of major proteins’ content in BM across different stages of lactation, with a secondary interest in exploring the impact of the mode of delivery on BM immune factors. This work was performed in China, a country presenting one of the highest rates of C-section birth in the world [28], and is part of the larger Maternal Infant Nutrition Growth (MING) initiative, conducted in a large cohort of urban Chinese mothers [29].

## 2. Materials and Methods

### 2.1. Subjects

This study was part of MING, a cross-sectional study designed to investigate the dietary and nutritional status of pregnant women, lactating mothers, infants and young children up to three years of age living in urban areas of China. In addition, the BM composition of Chinese lactating mothers was characterized for major proteins and immune factors. The study was conducted between October 2011 and February 2012. A multi-stage BM sampling from lactating mothers in three cities (Beijing, Suzhou and Guangzhou) was performed for BM characterization. In each city, two hospitals with maternal and child care units were selected and, at each site, mothers at lactation period 0–240 days were randomly selected based on child registration information. Subjects included in the period 0–5 days were recruited at the hospital whereas the other subjects were requested by phone to join the study; if participation was dismissed a replacement was made. Response rate was 52%. Recruitment and BM sampling, as well as baseline data collection, were done on separate days.

A stratified BM sampling of 540 lactating mothers in six lactation periods of 0–4, 5–11 and 12–30 days, and 1–2, 2–4 and 4–8 months were obtained in MING study. Nevertheless, only 450 BM samples were analysed in the present study, as the 0–4 days stage could not be included due to the limited volume of BM collected during this period.

### 2.2. Inclusion and Exclusion Criteria

Eligibility criteria included women between 18 and 45 years of age with singleton pregnancy, apparently healthy, full-term infant and exclusively breastfeeding at least until 4 months post-partum. Exclusion criteria included gestational diabetes, hypertension, cardiac diseases, acute communicable diseases and postpartum depression. Lactating women who had nipple or lacteal gland diseases, who had been receiving hormonal therapy during the three months preceding recruitment, or who had insufficient skills to understand study questionnaires were also excluded.

### 2.3. Ethical and Legal Considerations

The study was conducted according to the guidelines in the Declaration of Helsinki. All of the procedures involving human subjects were approved by the Medical Ethics Research Board of Peking University (No. IRB00001052-11042, 15-11-2011). Written informed consent was obtained from all subjects participating in the study. The study was also registered in ClinicalTrials.gov with identifier NCT01971671.

### 2.4. Data Collection

All mothers completed a general questionnaire including socio-economic and lifestyle aspects. Self-reported weight during pre-pregnancy and at delivery, number of gestational weeks at delivery, and delivery method were also recorded. Additionally, a physical examination evaluated basic anthropometric parameters (height, weight, mid-arm circumference) blood pressure and haemoglobin.

Data collection was done through face-to-face interviews, on the day of BM sample collection. In addition, date of birth and gender information of the infant was collected after the data collection since the data was not included in the initial questionnaires. Subjects were contacted by phone and were asked to clarify these two aspects retrospectively.

### 2.5. Sample Characteristics

BM sampling was standardized for all subjects and an electric pump (Horigen HNR/X-2108ZB, Xinhe Electrical Apparatuses Co., Ltd., Beijing, China) was used to sample the BM. Samples were collected at the second feeding in the morning (9–11 a.m.) to avoid circadian influence on the outcomes. Single full breast was emptied and an aliquot of 40 mL BM for each time point was secured for characterization purposes. The rest of the BM was returned to the mother for feeding to the infant. Each sample was distributed in 5 mL freezing tubes, labelled with subject number, stored at −80 °C and then shipped to the Nestlé Research Centre (Lausanne, Switzerland) for analyses within 6 months of collection.

### 2.6. Milk Sample Processing before Analyses

Frozen BM samples were skimmed by thawing to 4 °C, high speed centrifugation (2500× *g* for 10 min at 4 °C) and collection of the liquid fraction below the lipid phase. Each skimmed BM sample was then aliquoted in separate microtubes (Eppendorf AG, Hamburg, Germany) and frozen again until use. This aliquoting approach was put in place to avoid thawing-freezing cycles between the different analytical runs for the BM immune factors of interest as one aliquot was then dedicated to each analysis.

### 2.7. Measurement of Major Breast Milk Proteins

The following major BM proteins were measured in all 450 BM samples: α-lactalbumin, serum albumin, lactoferrin and all caseins. Due to the large number of samples, a classical approach using, for example, gel electrophoresis or HPLC separation did not provide sufficient throughput and speed. Therefore, an innovative microfluidic chip based quantitative method was specifically implemented and validated for BM protein analysis. The method was established on a LabChip GX-II instrument (Perkin Elmer, Waltham, MA, USA) allowing high-throughput analysis in a 96-well format. The principle of this technique is based on traditional SDS-PAGE protein separation but the whole procedure (separation, staining and detection) is integrated and fully automated in a microfluidic system. Results are provided in digital format (no gel staining or scanning, etc.). The general approach of this method was described previously [30] for bovine milk protein analysis and needed some slight adaptations for the BM sample analysis as described below.

#### 2.7.1. Sample Preparation

BM sample preparation was performed according to the LabChip (Perkin Elmer, Waltham, MA, USA) protocol. A simple 5-fold dilution of BM with water (Merck Lichrosolv quality) was found to be sufficient prior to protein denaturation and derivatization steps. In contrast to the immune factor analysis by ELISA, BM defatting was not required for the LabChip analysis thus avoiding potential protein losses. All sample preparation and processing steps were performed in 96-well format using electronic multichannel pipettes (Eppendorf Xplorer, Eppendorf AG, Hamburg, Germany). The HT Protein Express protein chip and reagent kit (Perkin Elmer, Waltham, MA, USA) was used for all analyses and highest purity reagents were required for all buffer preparations. Pure human milk proteins (α-lactalbumin, serum albumin, lactoferrin from Sigma, St. Louis, MO, USA) and bovine milk proteins (α-, β- and κ-casein from Sigma, as human proteins not available) were used as standards to generate individual calibration curves for each protein. The purity of each standard protein, according to the certificate of analysis, was used to calculate the true concentration of the protein standard in solution. Reported limit of detection of the LabChip system is 5 ng/µL according to the manufacturer. Calibration concentrations of the individual protein standards ranged from 25 to 750 ng/µL for serum albumin, from 50 to 1500 ng/µL for α-lactalbumin and lactoferrin, and from 100 to 3000 ng/µL for caseins. Note that as the individual casein proteins could not be fully resolved on the LabChip system, all casein peaks were integrated as one peak and thus one value for total casein concentration in BM was obtained (sum of α-, β- and κ-casein). In order to monitor system performance, a quality control sample (pooled BM from Lee Biosolutions Inc., Maryland Heights, MO, USA) was analyzed every 20th sample. All samples were analyzed in triplicates using a volume of 25 µL of BM.

#### 2.7.2. Method Validation

The method was validated for the determination of the four different proteins in human milk. For each protein (α-, β- and κ-caseins measured as total casein) the linear response of the LabChip detector was checked over the concentration range expected to be present in human milk samples. Each protein was analyzed at 8 different levels in triplicate. A quadratic regression was performed and linearity was assessed from the *r*^2^ and the plot of residuals.

To determine the trueness and precision of the method a milk sample was selected and spiked with the protein standards at 3 levels (the levels were adapted for each protein to cover the concentration range expected in milk). The non-spiked sample and the spiked samples were analyzed in duplicate on 6 different days. The spike experiments were used to determine recoveries, data from the duplicate analyses were used to determine repeatability (*r*) and data from the between day analyses were used to determine intermediate reproducibility (iR).

### 2.8. Measurement of Selected Breast Milk Immune Factors

Concentrations of IgA, IgG, IgM, TGF-β1 and TGF-β2 in BM samples were measured using selected commercial ELISA quantification kits that were specifically validated for their usage in milk matrix background. In more detail, IgA and IgG contents of BM were measured with Human IgA and IgG ELISA Kits from Bethyl Laboratories Inc., USA (Montgomery, TX, USA) (catalogue numbers E80-102 and E80-104, respectively), following manufacturer instructions and with milk samples tested at 1:20,000 and 1:1000 dilutions, respectively. Kit performance with such dilution factors were for IgA and IgG, respectively: average intra-plate repeatability 5% and 4.5%; average inter-plate repeatability 10.1% and 7.2%; average recovery 87% and 98%. IgM content was measured with the Human IgM Ready-SET-Go from Affimetrix eBioscience, USA (Santa Clara, CA, USA) (catalogue number 88-50620) following manufacturer instructions and with milk samples tested at 1:300 dilution. Kit performance with this dilution factor was: average intra-plate repeatability 3.8%; average inter-plate repeatability 4%; average recovery 90%. Finally, TGF-β1 and TGF-β2 contents were measured with Quantikine ELISA Human TGF-β1 and TGF-β2 Immunoassay Kits from R&D Systems, USA (Minneapolis, MN, USA) (catalogue numbers DB100B and DB250, respectively), following manufacturer instructions and with milk samples tested respectively at 1:5 and 1:4 dilutions on the top of the already 1:1.4 dilution of the original samples linked to the acidification and pH neutralization steps mandatory to activate latent TFG-βs from BM samples to their measured immune-reactive forms. Kit performance with such dilution factors were for TGF-β1 and TGF-β2 respectively: average intra-plate repeatability 4% and 9.3%; average inter-plate repeatability 6.1% and 10.8%; average recovery 84% and 92%.

### 2.9. Data Analysis

A multiple linear regression was applied to analyze the effect of lactation stage on the levels of the individual proteins. This model was adjusted for the effects of maternal age and BMI, infant gender, mode of delivery and geographical location.

A multiple regression model to explain the protein and immune parameter concentration was applied. The distribution of the residuals were checked via Box-Cox transformation method and a logarithmic transformation seemed to be adequate for all immune parameters. The following model was used:

log (concentration) = timeframe + sex + delivery + city + mother’s age + mother’s BMI + ε

The above model was the general model that was used to test for the effect of stage of lactation (timeframe) on immune parameter concentration taking in to consideration other variables such as gender, mode of delivery (natural vs. C-section) and geographic location (city). The term ε refers to a residual error (observed value–predicted value). With this model, contrast estimates were calculated comparing the successive timeframes (5–11 days vs. 12–30 days, 12–30 days vs. 1–2 months, etc.) to observe at which timeframes there were significant changes in nutrient concentration.

The same stage of lactation model was used in the subgroup of mothers delivering by C-section and also for natural delivery. In this case the model become simpler:

log (concentration) = timeframe + sex + city + mother’s age + mother’s BMI + ε

A similar model was used to assess the impact of mode of delivery with the difference of taking into account the interaction effect of time with the variable in question. The following model was used so that a comparison of the delivery modes can be made for each timeframe:

log (concentration) = timeframe × delivery + sex + city + mother’s age + mother’s BMI + ε

The same methods were used for the proteins, but a normality assumption is made and therefore no logarithmic transformation was performed on the 4 protein nutrients.

## 3. Results

### 3.1. Subject Characteristics

**Figure 1:**
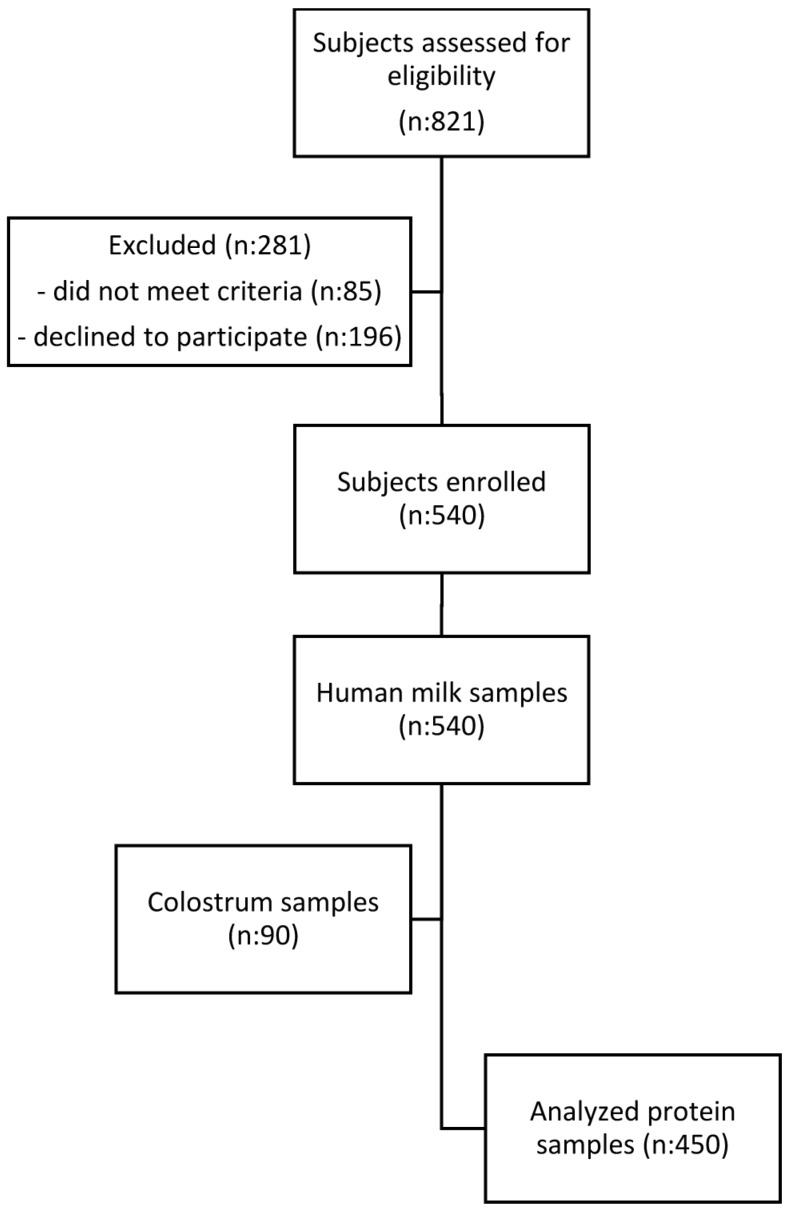
Study flow chart of subject recruitment.

In this cross-sectional study, nine different proteins were quantified in 450 BM samples collected at different stages from early to late lactation (8 months) in apparently healthy Chinese women from three different cities (i.e., Beijing, Guangzhou, and Suzhou). Figure 1 displays the recruitment flowchart from eligibility to sample analysis.

Subject demographics and anthropometry are described in Table 1. Maternal age, weight, body mass index (BMI) and mode of delivery were significantly different among the lactation stage cohorts. No other significant differences were observed in maternal and infant characteristics analysed. Note that the significant differences were taken into consideration for the analyses of protein contents at the different lactation stages as the statistical model was adjusted for these potential confounding factors.

### 3.2. Major Breast Milk Proteins

#### 3.2.1. Analytical Method Performance

**Figure 2:**
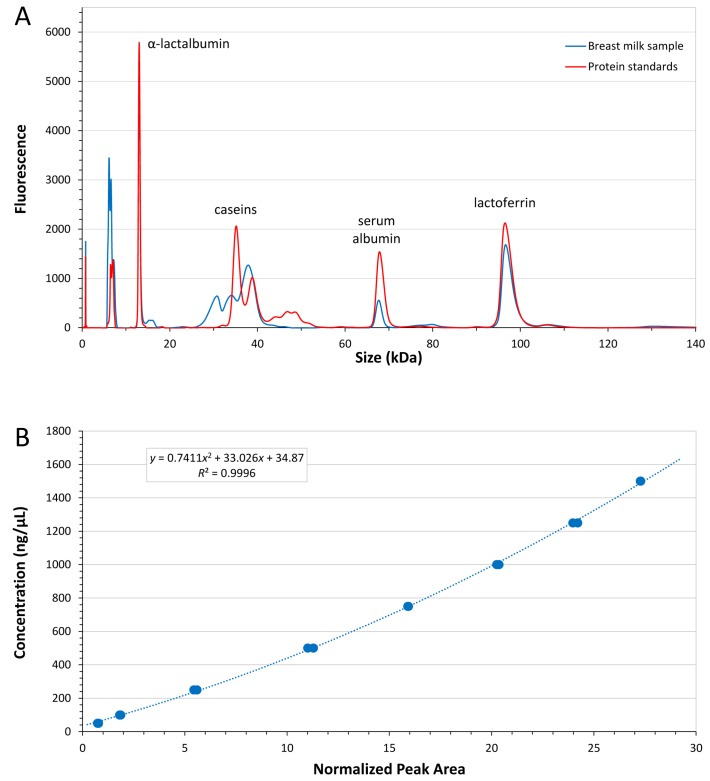
(**A**) Human breast milk protein separation on the LabChip GX II system. The electropherogram overlay depicts individual standard milk proteins (red) and a typical human breast milk sample trace (blue); (**B**) Calibration curve for α-lactalbumin (in duplicates, 50–1500 ng/µL, *R*^2^ 0.9996).

Each protein was quantified using an individual calibration curve (quadratic fitting, all *R*^2^ > 0.99, LabChip GX-II software, v4.1, 2015), based on a dilution series of pure standard proteins. Figure 2A depicts a typical electropherogram trace of a BM sample measured with the LabChip GX II system. A calibration curve for α-lactalbumin is shown in Figure 2B which demonstrates the small variation of replicate measurements. Based on a simple 5-fold dilution of the BM samples, limit of detection was 50 ng/µL for α-lactalbumin and serum albumin, 100 ng/µL for caseins and 130 ng/µL for lactoferrin, respectively.

The method was fully validated. Recoveries of proteins, determined using spiking experiments (three spiking levels, analyzed in duplicate on six different days), were between 91.8% and 116.5%. Relative repeatability (r%) for all proteins was <9.2% and relative intermediate reproducibility (iR%) was <26%. Measurement uncertainty was estimated using the simplified approach based on existing validation data proposed by Barwick [32]. The standard uncertainty (u) was determined at 11 ng/µL with relative standard uncertainty (u%) of 0.5%. Expanded uncertainty (U) was 19 ng/µL with relative expanded uncertainty (U%) of 1%.

#### 3.2.2. Analysis of Major Breast Milk Proteins

The concentration of α-lactalbumin decreased from 3.27 to 2.28 g/L over the investigated lactation period (Table 2). With the exception of the two first stages, all subsequent stages showed a significant decrease (*p* < 0.003) in α-lactalbumin content, over time until eight months. 

The concentration of lactoferrin also decreased over full lactation period, from 3.30 to 1.17 g/L (Table 2). This decrease was constant during lactation until 1–2 months, with significant differences (*p* < 0.000) in lactoferrin content between the first three investigated stages, and then stabilizing until the eighth months.

The concentration of serum albumin during the lactation period ranged from 0.48 to 0.42 g/L (Table 2) and did not show any significant differences between stages.

The concentration of caseins during the lactation period followed a different pattern than the other proteins, showing a significant transient increase from 5.84 g/L at stage 1 to 6.57 g/L in Stage 2 and 6.24 g/L in Stage 3 before returning to starting values in later stages (Table 2).

### 3.3. Breast Milk Immune Factors

IgA and IgM contents reflected the temporal change pattern of most of the major BM proteins with significantly higher contents in early milk before rapidly decreasing over time, reaching a basal plateau after 1 month of infant’s age (Table 2). IgG concentration did not follow the same pattern as it appeared stable throughout lactation (Table 2).

TGF-β1 and TGF-β2 BM contents proved to be also significantly higher in the very early milk (5–11 days period, Table 2). Then, while TGF-β1 concentration remained stable up to the end of the covered lactation period, TGF-β2 content continued to significantly decrease in the 1–2 months stage before significantly increasing again later back to 12–30 days levels.

**Figure 3:**
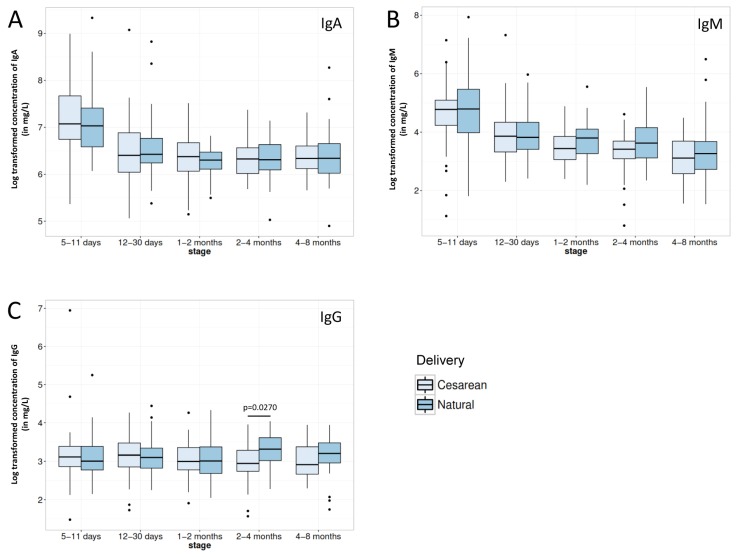
Comparison of (**A**) IgA; (**B**) IgM and (**C**) IgG immunoglobulin contents in breast milk from mothers delivering their infant either vaginally (Natural) or by Caesarean section for each lactation period of this study. Box plot represent medians with 25th and 75th percentile, min-max range and outliers. Statistical significance was set at *p* < 0.05 and significant *p*-values are indicated in the graphs.

**Figure 4:**
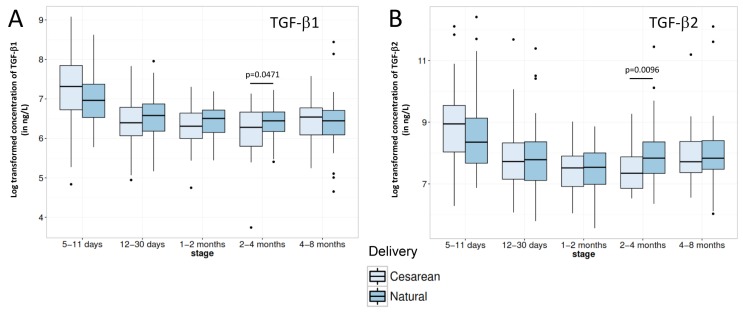
Comparison of (**A**) TGF-β1 and (**B**) TGF-β2 contents in breast milk from mothers delivering their infant either vaginally (Natural) or by Caesarean section for each lactation period of this study. Box plot represent medians with 25th and 75th percentile, min-max range and outliers. Statistical significance was set at *p* < 0.05 and significant *p*-values are indicated in the graphs.

Regarding impact of mode of delivery on immune factor contents in BM, even if occasional statistical significant differences or trends could be observed (see Figure 3 and Figure 4), C-section delivery did not appear to consistently impact BM concentrations in immune factor.

## 4. Discussion

### 4.1. Major Breast Milk Proteins

Various separation techniques and approaches have been exploited to identify and quantify milk proteins. Traditionally, chromatographic or electrophoretic methods have been used to profile major BM proteins [33,34,35] whereas immuno-based approaches, i.e., ELISA or antibody arrays, were the method of choice for quantitative analysis of individual proteins [36,37,38]. More recently, targeted LC-MS techniques have been developed to separate and quantify specific milk proteins [39]. The low throughput nature of these methodologies is often the limiting factor for application to larger numbers of samples. In order to address the challenge to precisely quantify major BM proteins in a large number of individual samples, as in the case of the present study, an innovative microfluidic chip-based method was specifically implemented, validated and applied in a high-throughput approach.

Despite a small limitation in the quantification of individual caseins, we believe that the minimal sample preparation and the 96-well sample format ideally combines speed and robustness of the analysis process and thus paves the way for a new technological standard for the measurement of α-lactalbumin, lactoferrin, serum albumin and total caseins in future studies addressing BM protein composition.

α-lactalbumin was the most abundant whey protein in the Chinese mother’s BM samples (Table 2), in agreement with previous data in the literature [19]. Even though its content decreased significantly over the first two months, it remained high in all lactation stages. The high levels of this protein in BM are likely key for the nutrition of the breast-fed infant. Contrary to other BM proteins, α-lactalbumin appears to be fully hydrolysed and absorbed in the infant intestine, thus to be a good source of nitrogen and indispensable amino acids [40]. This protein contributes as well to the balanced amino acid composition of BM and, in particular, to its high levels of tryptophan, which ultimately allows BM to cover the infant’s amino acid requirements with limited amounts of protein [41]. High levels of tryptophan in this protein would be also associated with its protective effects against epileptic seizures in animal models [42]. Besides its nutritional role, the α-lactalbumin HAMLET complex found in BM has anti-tumoral [9] and bactericidal effects [8]. Furthermore, α-lactalbumin bears bioactive peptide sequences (e.g., mineral chelator or anti-microbial peptides) that may be released and transiently exert their activity (e.g., increased mineral absorption or prevention of infection) in the infant intestine. In line with this, Kelleher et al. [43] observed increased mineral absorption and Bruck et al. [44] inhibition of *E. coli*-induced diarrhoea in infant monkeys fed a formula supplemented with bovine α-lactalbumin.

The second most abundant whey protein in our samples was lactoferrin (Table 2) for which the observed concentration values and gradual decrease along lactation were similar to those previously reported [19,45]. The bioavailability of lactoferrin, thus its nutritional relevance as a source of amino acids and nitrogen to the infant, is not known. However, it has been found intact in infant faeces and resistant to digestion in an in vitro digestion model, which suggest that the nutritional role of lactoferrin may need reconsideration and further studies [40]. In contrast, multiple biological activities have been proposed for this protein [40] including infant protection against gastrointestinal infection and sepsis [7].

It is noteworthy that temporal changes trends of α-lactalbumin and lactoferrin along lactation are fully aligned with that of the total proteins measured in the same BM samples as previously reported [29].

Due to the complexity of the casein composition in BM and limitations in mass resolution of the new analytical method used in this study, caseins were not quantified individually but as total caseins (combining α-, β- and κ-casein concentrations, Table 2). This novel analytical approach, however, was potentially susceptible to introducing some bias in comparison to the more classical acid-precipitation based methodology. Interestingly, our results demonstrated that this was not the case as the increase of casein concentration at the beginning of lactation followed by a slight decrease at the later period was closely aligned with a previous report [46]. These results support the theory that casein and whey protein synthesis and/or secretion is regulated by different mechanisms in the mammary gland [47].

In contrast to the other major whey proteins, serum albumin concentration stayed mostly constant across the lactation stages. Comparison with literature data showed similar concentrations in our samples [19]. It is noteworthy that, contrary to other BM proteins that are synthesized by the mammary gland, serum albumin is transferred from the maternal blood [2]. To our knowledge, no specific biological activities have been attributed to this protein. It certainly contributes to the nutrition of the infant as it appears to be highly digestible [2].

### 4.2. Breast Milk Immune Factors

BM contents of immune factors at the different lactation stages (Table 2) were also in agreement with the previously published ranges [18,48,49,50,51], as well as with our own previous findings (unpublished data), indicating that BM from Chinese mothers does not differ from worldwide references in these bioactive components.

IgA and IgM BM contents were significantly high in early milk before rapidly decreasing over time, reaching a basal plateau after 1 month of infant’s age, while IgG content was stable throughout lactation. These differences in production pattern between IgG and both other immunoglobulins resides in the fact that IgA and IgM are actively secreted in BM through the poly-immunoglobulin receptor expressed by mammary gland epithelial cells [12] while IgG is more passively appearing in BM through transudation from the systemic circulation, as already mentioned above for serum albumin.

While TGF-β1 contents of BM followed the same temporal changes that the majority of investigated proteins as previously described [21], variation of TGF-β2 concentration appeared to be slightly different, also with a strong decrease until the 1–2 months lactation stage but followed by an increase, reaching at 8 months the level observed in the second half of the first month of lactation (Table 2). Such type of fluctuating pattern of TGF-β2 BM content has already been observed in a previous study [49], however, to a smaller extent and at different time points. Whether this evolution of TGF-β2 has a physiological role for the infants remains to be determined. Indeed, studies mainly focusing on TFG-β1 and 2 demonstrated that milk-borne TGF-βs regulate inflammation [13], stimulation of IgA isotype switching in B cells [52], maintenance of intestinal epithelium barrier function [14], induction of oral tolerance [15], and consequently help to prevent allergic diseases [53,54].

It is noteworthy that C-section delivery did not appear to consistently impact BM content in the major immune factors measured in the present study. The high number of mothers participating in our study together with the high rate of C-section delivery thus allows increasing knowledge on the previously described limited impact of the mode of delivery on major immune BM proteins [4,27], while extending at the same time the former observations on colostrum to transitional and mature milk. Moreover, our data also tend to indicate that the increased risk of immune-related diseases associated with C-section delivery may not be associated with any alterations of major BM immune factors’ composition. However, we cannot rule out a potential effect of the mode of delivery on the immune factor composition of the colostrum as our earliest milk samples were collected 5–11 days after delivery. In addition, the impact of delayed onset of lactation following C-section [25,26] on later infant health was not assessed in the present study and would deserve deeper investigation in order to further consolidate the above hypothesis.

The cross-sectional nature of our study limits the conclusions related to the stage-driven changes, which would have been best assessed by a longitudinal design. However, our statistical model adjusted for the maternal and infant baseline factors known or suspected to impact on milk nutrient composition [55]. Our results are also reinforced by the fact that they were remarkably consistent with those previously published.

## 5. Conclusions

This multi-centric cross-sectional study covering 8 months of lactation for 450 Chinese mothers demonstrated that their BM content in major proteins was comparable with previous studies carried out in other parts of the world, highlighting that key protein components of BM are conserved across geographic localization. Moreover, this study is to our knowledge the first one to address the effect of C-section delivery on major BM immune factors throughout transitional and mature milk, actually showing that C-section delivery had very limited impact on the maternal-to-offspring transmission of active immune competence.

## Figures and Tables

**Table 1:** Maternal and infant characteristics (adapted from [31]).

Study Population	5–11 Days	12–30 Days	1–2 Months	2–4 Months	4–8 Months
	(*n* = 90)	(*n* = 90)	(*n* = 90)	(*n* = 90)	(*n* = 90)
**Mother**					
Age (years), Mean (SD)	27 (4)	27 (3)	28 (4)	27 (4)	26 (4)
Height (cm), Mean (SD)	160 (4)	160 (5)	161 (5)	161 (5)	159 (5)
Weight (kg), Mean (SD)	60.7 (8.7)	60.8 (7.9)	61.9 (8.9)	58.4 (8.3)	56.2 (8.1)
BMI (kg/m^2^), Mean (SD)	23.7 (3.3)	23.7 (2.8)	23.9 (3.1)	22.5 (2.9)	22.2 (3.1)
Gestational weight gain (kg), Mean (SD)	16.7 (7.4)	16.2 (6.0)	15.9 (5.7)	15.9 (5.9)	14.9 (7.6)
Postpartum weight loss (kg), Mean (SD)	9.1 (6.1)	8.6 (5.3)	9.8 (4.0)	10.0 (6.2)	10.6 (5.9)
Caesarean delivery, *N* (%)	39 (42)	43 (48)	53 (59)	35 (39)	35 (38)
**Infant**					
Males, *N* (%)	51 (57)	48 (53)	48 (53)	54 (60)	43 (48)
Gestational age at birth (weeks), Mean (SD)	39.3 (1.2)	39.2 (1.3)	39.2 (1.6)	39.4 (1.3)	39.5 (1.5)

**Table 2:** Protein content of human breast milk from the different lactation stages (see also Figure S1).

Proteins	5–11 Days	12–30 Days	1–2 Months	2–4 Months	4–8 Months
	(*n* = 90)	(*n* = 90)	(*n* = 90)	(*n* = 90)	(*n* = 90)
**Major breast milk proteins**					
α-lactalbumin (g/L), Median (IQR)	3.27 (0.60)	3.16 (0.55)	2.84 ^a^ (0.55)	2.53 ^a^ (0.47)	2.28 ^a^ (0.63)
Lactoferrin (g/L), Median (IQR)	3.30 (2.11)	1.86 ^a^ (0.89)	1.24 ^a^ (0.53)	1.15 (0.46)	1.17 (0.47)
Serum albumin (g/L), Median (IQR)	0.48 (0.14)	0.48 (0.14)	0.42 (0.09)	0.44 (0.10)	0.42 (0.08)
Total caseins (g/L), Median (IQR)	5.84 (3.17)	6.57 ^a^ (2.15)	6.24 (2.25)	5.79 ^a^ (1.69)	5.60 (1.73)
**Immune factors**					
IgA (mg/L), Median (IQR)	1148 (1022)	615 ^a^ (494)	553 ^a^ (232)	557 (312)	564 (337)
IgM (mg/L), Median (IQR)	117 (168)	47 ^a^ (47)	35 ^a^ (31)	35 (29)	25 ^a^ (25)
IgG (mg/L), Median (IQR)	22 (13)	23 (12)	20 (14)	24 (15)	23 (14)
TGF-β1 (ng/L), Median (IQR)	1258 (1305)	685 ^a^ (482)	600 (356)	598 (379)	659 (410)
TGF-β2 (ng/L), Median (IQR)	5286 (10,444)	2322 ^a^ (3100)	1877 ^a^ (1890)	1920 ^a^ (2112)	2311 ^b^ (2868)

^a^ *p* < 0.05 vs. previous stage; ^b^ *p* < 0.05 vs. previous 1–2 months stage.

## Supplementary Materials

The following are available online at http://www.mdpi.com/2072-6643/8/8/504/s1, Figure S1: Comparison of major milk protein content for (A) α-lactalbumin; (B) lactoferrin; (C) serum albumin and (D) total casein for each lactation period. Box plot represent medians (*n* = 90 per lactation period) with 25th and 75th percentile, min-max range and outliers. Statistical significance was set at *p* < 0.05 and significant *p*-values are indicated in the graphs.
