# Supplementary material for: Temporal Changes of Protein Composition in Breast Milk of Chinese Urban Mothers and Impact of Caesarean Section Delivery
Source: Nutrients. 2016 Aug 17;8(8):504. doi: 10.3390/nu8080504 (PMC4997417; doi:10.3390/nu8080504)
Supplement: Supplementary file 1 [file nutrients-08-00504-s001.docx]

Supplementary Materials: Temporal Changes of Protein Composition in Breast Milk of Chinese Urban Mothers and Impact of Caesarean Section Delivery

Michael Affolter, Clara L. Garcia-Rodenas, Gerard Vinyes-Pares, Rosemarie Jenni, Iris Roggero, Ornella Avanti-Nigro, Carlos Antonio de Castro, Ai Zhao, Yumei Zhang, Peiyu Wang,
Sagar K. Thakkar and Laurent Favre


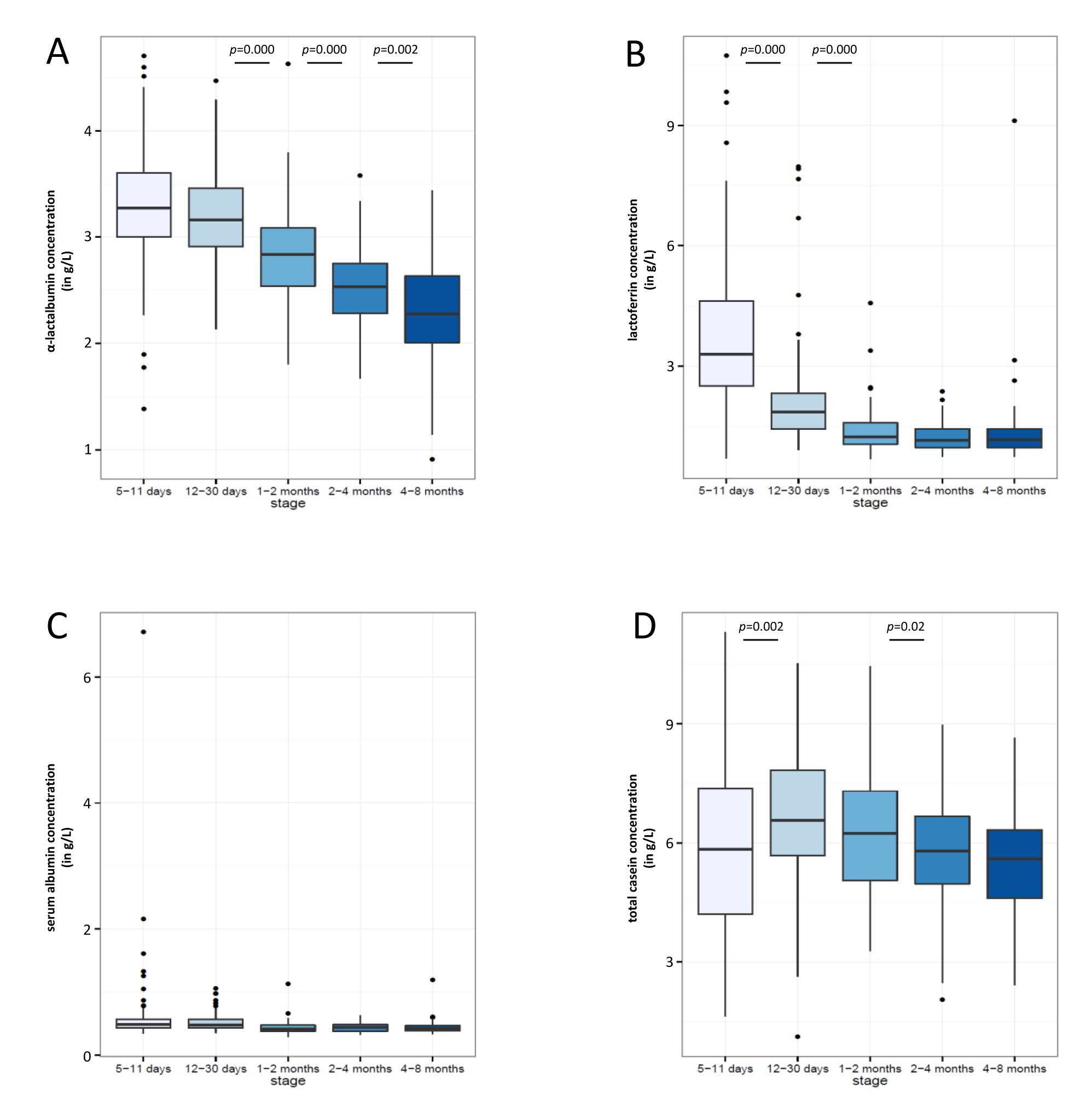


**Figure S1.** Comparison of major milk protein content for (**A**) α-lactalbumin; (**B**) lactoferrin; (**C**) serum albumin and (**D**) total casein for each lactation period. Box plot represent medians (*n* = 90 per lactation period) with 25th and 75th percentile, min-max range and outliers. Statistical significance was set at *p* < 0.05 and significant *p*-values are indicated in the graphs.
